# Is moral adequacy possible in the face of structural disadvantage? The experiences of health and social care staff in supporting homeless people using substances at the end of life

**DOI:** 10.1177/26323524251382262

**Published:** 2025-11-26

**Authors:** Gary Witham, Gemma Anne Yarwood, Sarah Galvani, Lucy Webb, Sam Wright

**Affiliations:** 1 School of Nursing and Public Health, Manchester Metropolitan University, UK

**Keywords:** substance use, palliative care, homelessness, moral distress

## Abstract

**Background:**

Homeless people using substances at the end-of-life face many challenges in accessing and receiving good care. These can relate to poor interdisciplinary working by health and social care practitioners, stigma and structural disadvantage.

**Objective:**

Using positioning theory, we explored the challenges of existing models of practice for practitioners supporting this population.

**Research design:**

This is a qualitative descriptive study in which data were collected via four interdisciplinary practitioner focus groups.

**Data sources and methods:**

The four online interdisciplinary focus groups were conducted within a region of North-West England. This included 24 participants from health and social care providers with experience of working with and/or supporting people experiencing homelessness and using substances.

**Results:**

The findings indicated three primary discourse positions related to (i) What constitutes a good death and where? (ii) The limitations of professional boundaries and (iii) Maintaining moral adequacy in the face of traumatic death. For practitioners, maintaining moral adequacy was often compromised by ineffective multi-disciplinary collaboration. Practitioners were often exposed to traumatic working experiences with limited resources to effect change.

**Conclusion:**

The findings support work examining the structural and environmental challenges of palliative care provision for hostel-users and unsheltered homeless people in providing care at the end of life for people experiencing homelessness.

## Background

Interdisciplinary working by practitioners can be challenging within the context of stigma and structural disadvantage experienced by homeless people who use substances at the end of life.^1–3^ While there is a growing evidence base examining professionals’ experiences of responding to homeless people at the end of life,^4–7^ there is a scarcity of research focusing solely on professionals’ experiences of supporting people using substances (alcohol and other drugs (AODs) who have end-of-life care needs, a subgroup of whom are homeless. Shulman et al.^5^ explored the challenges in the provision of, and access to, palliative care for people who were homeless. They sought the experiences of homeless people and health and social care staff using a short, constructed scenario (vignette) of someone with alcoholic liver disease within interviews and focus groups. They described the inexperience of many practitioners in working with people who are homeless and using AODs and who present with challenging behaviors. They found fragmentation and limited coordination between Housing, Health and Social Services, often preventing a more person-centered approach to care. They also found that living with chronic diseases could mean that prognosis was uncertain, leading to a delay in timely referrals to appropriate palliative care services. Such delays can be compounded by current narratives of “recovery-focused” care prevalent within UK drug and alcohol services.^8^ This recovery focus, underpinned by a drive for abstinence can marginalize risk reduction strategies and discourage social and healthcare professionals from reflecting on their ability to support people to die well while taking substances.^9–12^ In such contexts, talk about death and dying can be silenced, and this can extend to people using substances who may be reluctant to take opioids for pain management at the end of life. They may have worked hard to maintain recovery and requiring opioids could be perceived as a failure, undoing the work leading to this point.^13^

Stajduhar et al.^4^ in their ethnographic study reported the challenges of identifying people who were homeless at the end of life who also used substances. This population may benefit from a palliative approach, however, the barriers to accessing care and the conditions people are living with make both the identification of serious and advancing ill-health and the need for palliative care, challenging to identify. Stajduhar et al.^4^ highlighted how people’s focus was on basic survival needs leaving little space to explore preferences related to end-of-life care. In addition, stigmatization by formal care providers restricted access to care, for example, a lack of home support related to end-of-life care if that space was deemed unsafe, such as shelter accommodation.

Stigma and discrimination often leave homeless people using AOD reluctant to search for, or accept care from, formalized palliative services.^14,15^ As a result of waiting until the symptom burden is too great to self-manage, people who use AOD typically present to healthcare services very late, which leads to emergency provision rather than appropriate advance care planning.^16^ Hospice care for this population may be viewed by other professionals with misgivings: perceived as inappropriate for someone using AOD, or a place of no return that isolates people from street friends and other support networks.^6^ From the hospice perspective, there can be challenges relating to anti-social behavior associated with AOD use with potential diversion of prescribed opioids to family or friends or the risk of them bringing illicit drugs into the hospice.^17^ Coupled with poor interdisciplinary communication and limited palliative care training for hostel or shelter staff, there were often delays to people receiving interventions that could provide access to end-of-life care.^6,18^

This article reports on findings from four focus groups with front-line professionals from social care, health, AOD use and housing services with a particular focus on homelessness. It builds on previous research by focusing on people using AOD, examining existing models of practice and professionals’ care experiences in greater depth.^19^ This study also builds on research related to moral distress in the context of homelessness with data from Morris et al.^20^ highlighting the complexity of client need, systemic barriers in offering support and resource gaps all negatively impacting on social workers. Johnson et al.^21^ further explored, in their interview data, the experiences of moral distress for health and social care practitioners. There was a disconnect between expectations versus reality when working in practice with feelings of helplessness given limited resources. Moral distress appeared to generate a disconnect with clients leading to avoidant behavior in communication that affected decision-making. Connecting with allies within an organization and wider community was a strategy used to manage institutional restraints that often led to moral distress.

In terms of the concepts and definitions used within this article, structural disadvantage can be defined as either an individual’s or population’s risk of poorer health outcomes based on normative socioeconomic, political and cultural hierarchies.^22^ Moral adequacy is framed within the context of “moral distress,” a concept introduced by Jameton^23^ in which “moral distress arises when one knows the right thing to do, but institutional constraints make it nearly impossible to pursue the right course of action” (p. 6). Health and social care practitioners have a duty of care but can be presented with situations that challenge their perception of the morally right course of action.^24^ Moral adequacy refers to the extent to which an individual’s actions align with their moral principles and societal expectations. Within this paper, moral adequacy is operationalized in examining the positioning of moral principles with social expectations.^25^ This approach has been employed within the context of both advanced dementia and people using substances at the end of life.^12,26^ To maintain moral adequacy, there is an obligation to care and support structurally disadvantaged communities who are vulnerable but practically this is not always possible and can lead to moral distress.

## Research question/aim/objectives

The aim of this paper is to explore the ethical challenges professionals face within existing models of practice. One of the primary objectives, addressed in this paper, is to determine the experiences, resource and support needs of the health and social care professionals caring for people who experience homelessness and use AOD with end-of-life care needs.

### Research design

This study used a qualitative approach exploring the experiences of health and social care practitioners through conducting four interdisciplinary focus groups. Focus groups offered the opportunity to capture diverse perspectives by bringing together practitioners in a discursive research group context.

### Participants and research context

Participants were recruited within a region of North-West England. All the participants had experience of working with and/or supporting people using AODs. Participants were purposively recruited from 10 partner organizations, including hospice and palliative care services, charities, mental health services and specialist social care (Table 1). They were recruited via the lead contacts in each partner agency who disseminated participant information sheets on the project to their staff group. Staff who wished to participate could contact the research team for further information. A research poster, highlighting the research project, was also disseminated through notice boards and offices of partner organizations. In terms of methodology, four focus groups were conducted, with Guest et al.^27^ suggesting that 90% of all themes can be generated within three to six focus groups. The sampling of participants in each focus group (Table 1) represented interdisciplinary diversity to present a range of positions and perspectives related to the complexity of supporting people using AOD at the end of life.

**Table 1.** Focus group characteristics.

Focus group	Participant numbers	Practitioner role
Focus group 1	7	Social worker (*n* = 3)
		Hostel manager (*n* = 3)
		Regional palliative care manager (*n* = 1)
Focus group 2	5	Social worker (*n* = 2)
		Palliative care register (*n* = 1)
		Hostel manager/worker (*n* = 2)
Focus group 3	8	Social worker (*n* = 3)
		Hospice nurse (*n* = 1)
		General practitioner (*n* = 1)
		Substance misuse worker (*n* = 3)
Focus group 4	4	Hostel manager/worker (*n* = 2)
		Hospice nurse (*n* = 2)

The sample consisted of 24 participants (see Table 1), comprising 17 female and 6 male health and social care professionals. Only 10 participants returned demographic questionnaires, among whom 9 were White British and 1 Black British.

An interview guide was developed by the whole research team prior to gaining UK National Health Service Health Research Authority ethical approval for the project and was used to explore models of good practice (Table 2). This focused on the participants’ current model of care and the challenges experienced personally and professionally in supporting this population. Finally, we sought to establish the kind of training and support that enabled effective working with people using AODs at the end of life. All the focus groups were conducted by one researcher between October 2020 and February 2021. They were held online using Microsoft Teams Software due to the COVID-19 pandemic prohibiting face-to-face contact. They were recorded (with permission) and transcribed verbatim. All the focus groups were facilitated by the same researcher to both maintain consistency and gain insights and perspectives that could potentially be explored in the other groups.

**Table 2.** Interview guide.

Topic Guide [subsequent questions are prompts]
1. What is your organization’s current practice when someone who uses/has used substances has a terminal illness? a To what extent does this happen? b What is your organization’s experience in delivering this? *[PROMPT for any limitations]* c What is your experience in practice? d To what extent is asking about substance use and terminal illness part of routine questioning and assessment for people entering the service? Who does this? e What support is available for family caregivers? 2. What support have you had for working with this group of people? a How supported do you feel in working with (a) the person themselves or (b) family members in these contexts? b What support do you need? c It can be difficult for practitioners to talk about EoL care and/or substance use – how can they best be supported to overcome this? d What might help you feel more supported? 3. What does good quality end of life care look like for people who use/have used substances? a How could we improve local palliative/ End of Life Care (EoLC) for people who use/have used substances? b How can we improve current systems or pathways to enhance care quality? c What might a model of care look like locally in terms of palliative or eol care for people using substances? d Where are the gaps in current care delivery models, if any? How could they be addressed? e If resources were no object what might it look like? [*Prompt if needed*] 4. What barriers to accessing EoLC exist for people who use/have used substances and who currently have little or no involvement with services? a Who is currently missing from EoLC provision? b How can we improve access to services for these people? *[Prompt for both practice and policy/commissioning change]* c How could a new model of care help those people to access EoL or social care? d What resources are available to support this work? How could any resource constraints be overcome? 5. To what extent is it possible currently to deliver joined up care between substance use and palliative/end of life care services? a Why is that? What helps/hinders joined up care? b How could a new model of care improve this? c What support could be incorporated into a new model of care to help practitioners maximize joined up working across substance use, EoLC, health and social care services? d Which other services need to be centrally involved in this work? And which ones are currently missing? 6. What sort of training have you and your colleagues had to date on working with people at end of life who use/have used substances? a What sort of training do you think would be helpful for you, your colleagues and your organization under this new model? b What do you feel you need to know in order to work effectively in these situations? c Did the training cover the breadth of people who come into this group? Was that helpful? d Do you have any training needs relating to assessment processes/routine questioning? 7. Any other comments you would like to make or information you would like to share?

### Ethical considerations

Ethical approval was sought and granted from the Health Research Authority and Health and Care Research Wales (REC reference: 20/WM/0140, June 9, 2020). Participants were recruited voluntarily, and all verbal and written informed consent was obtained prior to the focus groups. Privacy and confidentiality were prioritized using pseudonyms and secure data storage methods.

### Analysis

The four focus groups were analyzed using positioning analysis.^28^ Positioning analysis is a systematic examination of the interaction between people, locating both how the person identifies within an interaction and how that relates to the normative cultural framing of such interactions.^29,30^ It is underpinned by positioning theory, which is grounded in everyday conversation and involves teller and listener negotiating the construction(s) (or attempted construction(s)) of action related to social practice. The theory addresses features relating to the local context of the interaction within a focus group and highlights the explicit and implicit patterns of reasoning that are generated in the ways that people act toward each other. This approach supports a critical analytical framing of interaction in, for example, focus groups where participants relate to each other through multiple positionings, for example, through their job role or professional affiliations.^31^

The transcripts from the four focus groups were uploaded to NVivo 11 software to support data analysis. The process of positioning analysis involved:

1. Systematic reading of the four focus group transcripts.
2. Identification of the lived storylines that the participants adopted. This included their background and history as well as the ongoing interaction within the focus group.^31^
3. Identification and annotation of speech acts (e.g., questions/comments) where practitioners performatively assign and adopt positions in the interaction. We focused on how the participants spoke about their practitioner duties, including how they maintain moral adequacy within the specific storylines generated within the focus group.
4. Iterative highlighting of common constructions and discursive positions focusing on the challenges of practice in supporting people using AODs to all four focus groups.

The positions of different health and social care practitioners were initially developed by one of the research team and then independently reviewed by a second team member for rigor, accuracy and quality control purposes. Each researcher had either a health or social care background, significantly enabling a greater insight into the positioning of the participants. These data were generated through a prolonged engagement with participants in the context of a wider project. This allowed sufficient time with participants and the research context to build trust, rapport and honesty within the focus groups. Member checking^32^ was conducted in real time in each focus group and involved the interviewer presenting any tentatively identified issues to the participants for confirmation or clarification. To embed rigor, this process was also developed across focus groups with the researcher, where appropriate, drawing out and presenting initial findings from the other focus groups.

## Results

The data analysis resulted in three key discourse positions relating to how practitioners position themselves in relation to the practice challenges of supporting homeless people using AODs and approaching end of life. These were as follows: (i) what constitutes a good death and where, (ii) the limitations of professional boundaries and (iii) maintaining moral adequacy in the face of traumatic death.

### What constitutes a good death and where?

A good death has been characterized as featuring elements of control, and a death that is in keeping with what that person wishes, both can be compromised by a denial of dying and uncertainty in identifying or acknowledging dying.^33^ There was consensus amongst participants that a good death presented a challenge and may not be prioritized for people who are homeless and using AOD at the end of life. For example, a hostel worker presented a narrative of a client using AOD who was dying. What he positioned as challenging was the client’s apparent self-neglect whereby the focus was acquiring alcohol or/and illicit substances to the detriment of his health. The hostel worker identified the AOD use as “self-neglect” rather than perceiving this as a potential form of self-care whereby the person self-medicated to manage distress or symptoms of poor mental health:

So, when we were informed he was on an end-of-life programme. . ., it was very tragic really. I mean it was a lot of self-neglect and abuse in-between that time, and things I’ve never seen, the sort of medical condition that he was nil-by-mouth, so he had no esophagus and stuff like that. It was really sad, the physical but also the mental health decline too, knowing his life was restricted heavily and stuff like that. (Hostel worker FG 1)

However, the quote also depicts the tragedy and sadness of his client’s dying in a context that places hostel staff at the epicenter of managing this complexity on a daily basis. Some staff felt such choices were difficult to understand, but were aware of the consequences of addiction and the reluctance of clients to access support, as a support manager commented:

if you’ve got somebody that’s got an addiction, that addiction is not just going to go away so they all need to find a way to accommodate that addiction, regardless of the setting really in these circumstances. I mean this has an effect on all of our residents you know a lot of the reasons why their health issues are so bad is because they don’t engage in support. Because they won’t engage in the support because it means they have to go away from their drugs. (Support manager FG 4)

There were also the difficulties of finding suitable accommodation, and this was a significant issue for participants, particularly those working in housing and social care. They positioned social care providers as offering limited adequate care, not just in terms of staff practice but also within the facilities and buildings in operation, as well as the structural and environmental context. Existing accommodation remained unsuitable for complex physical care, for example, with no space for hoists or with single bathroom facilities. This led some participants working in hostel and temporary housing to suggest specialist hostel accommodation with dedicated end-of-life beds. Identifying suitable end-of-life accommodation was positioned as a difficult task for participants, situated and exacerbated in systems, rules and protocols of hospices or hostels that often-restricted continued AOD use.

The lack of engagement with formalized services by people using AOD was highlighted by a participant working in harm reduction services. This reluctance to engage with services by people using AOD originates from their previous experiences of stigma and discrimination. This results in people being cautious and reticent about help-seeking. In turn, their absence from services can also lead to decision-making that excludes the person who is homeless and using AOD. Providing a suitable care environment while trying to maintain person-centered approaches to care was a considerable challenge for the majority of participants. Without the person’s engagement, and in the presence of deteriorating health, there were limitations on the “best interest” decisions participants could make.

Particularly for social care practitioners, stigma and discrimination were positioned as a challenge for their clients in respect of suitable accommodation. This was contextualized in examples of anti-social behavior. One social worker commented on a client’s behavior in supported housing:

Oh, their behavior, they want them to move somewhere else, So I’m ending up being involved in the MDT [multi-disciplinary team] as well because I have to say, ‘Well the behavior, when does it come? It shows up when the person is drinking. Once they’re sober, they’re fine, they’re cooperating, they follow the rules, it’s that time when they’re drinking.’ But to the supportive accommodation, there’s other people there, so it’s like, ‘they’re disruptive’ I’m like, ‘Well you can’t move them, you can’t just kick them out because it’s a disease.’ (Social worker FG 2)

The social worker used reported speech to explore the dilemma of disruptive behavior relating to alcohol use. She positions her dialogue to challenge the decision to remove the client from the sheltered housing by quantifying the extent and time of the behavior and questions; where are they going to go? There is an ethical challenge presented at the end, this client is “ill” with an addiction and remains potentially marginalized if removed from the supported housing.

Hostel staff also described the challenges and fears of clients in engaging with health-related services due to previous negative interactions. This fear was often further exacerbated by policies that remain inflexible, for example, a participant (a palliative care consultant) positioned hospices in a way that “others” people using AOD at the end of life. She presented operational aspects of hospices as challenging in addressing patient complexity, citing inadequate training leading to compromised care. This participant also referred to a lack of freedom in hospices with doors often locked at night. This again highlights the environmental and structural barriers to appropriate end-of-life care for people who are homeless using AODs. Restricting access and limiting freedom may create mistrust and suspicion given an historical context in which homeless and AOD using populations often experience stigmatization and discrimination from formalized healthcare providers.

A palliative care consultant (FG3) also positioned anti-social behavior as very distressing to other hospice residents and family:

. . . so occasionally, it becomes what’s going to be the best for the greater good unfortunately and sometimes that’s when we have to say we can’t keep on providing care [in the hospice] for someone who desperately needs it because we’ve got a building full of other patients and families who are being significantly negatively impacted by someone. (Palliative care consultant FG 3)

In addition, there may be a fear of medicine diversion, where friends or family may try to take opiates from residents using AODs at the end of life to use or sell. There may also be potential episodes of aggressive or disrupted behavior related to access restrictions to AOD.

The palliative care consultant presented a utilitarian position (an ethical position asserting we ought to act to produce the greatest good for the greatest number) to justify her ethical stance while acknowledging the needs of someone using AOD. She contrasts this “desperate” need of one individual within a full hospice of other patients and families. This highlights the real challenges of meeting the complex needs of this population within a traditional hospice environment.

### The limitations of professional boundaries

Significantly, the interdisciplinary nature of these focus groups meant there was a clear positioning from participants as they used speech acts to reference their professional backgrounds and position this within the talk about the challenges related to this population. There remained a demarcation between health and social care with professional policies, guidelines and boundaries affecting care responsibilities. This further displayed the structural complexities of navigating different systems between social care and health. The participants presented stories illustrating both communication and service gaps relating to the ambiguity of who should be responsible in providing support for people using AOD at the end of life. The complexity of care was highlighted by participants in working with this population, and this included the difficult, coordinated, multiagency, collaboration required.

Complexity of care was often presented by narratives relating to personal care and medication management, highlighting the complex UK regulatory requirements for hostel and temporary housing providers, particularly in reference to medicines administration and management. Such concerns highlighted questions about risk, responsibility and professional boundaries:

. . . it was very confusing about what we could and couldn’t do with the barriers of CQC [Care Quality Commission] and medication and things like that. It was quite frantic the first time that it happened, it was quite scary for us and the other residents. (Hostel support worker FG 1)

The hostel worker positions her whole team as finding it challenging to support someone using AOD at the end of life while remaining within the regulatory requirements for medicines or other more health-oriented interventions. The response of this initial problem was characterized as frantic and frightening for staff and other residents. Hostel staff had to try to recognize dying early to make timely referrals to specialist palliative care services for both medicine management and personal care. The challenge of recognizing end of life was highlighted by hostel staff with client co-morbidities often having an impact on people who were homeless and using AOD. The accumulated effect of chronic conditions may be that staff are slow to recognize or acknowledge end of life, particularly with limited medical training, as one participant commenting:

I suppose, I mean, not having any medical background but thinking about the chronic COPD, the asthma, the blood-borne viruses, the various other infections that are constantly present, might not actually lead them to have the actual diagnosis [i.e. end of life] but I suspect they’re on that pathway anyway. So, it can be a bit tricky I think because of that. (Hostel worker FG 4)

This hostel worker presents her professional boundaries to the group, acknowledging her limited medical knowledge. However, she then names and categorizes a series of chronic conditions that could affect her clients, even if there was no formal diagnosis. This nuanced interpretation underlines a clear understanding of the implications of medical conditions and the consequences that may impact on her clients. Hostel workers often recognized the complexity of dying but this does not necessarily make the pathway to collaborative working with palliative services any more straightforward. Having the confidence to formally identify deterioration in a client’s condition and effectively signpost to palliative care services was challenging for hostel workers.

There was participant consensus for those who discussed the significant problem of practitioner outreach to structurally disadvantaged populations. This appeared less to do with beneficial outcomes of delivering outreach but rather the complexity of care with potential interdisciplinary involvement, meaning that sharing expertise was significantly more challenging given the potential number of agencies involved. The challenges of outreach were also exacerbated by client attitudes to healthcare services based on poor prior experiences of stigma and discrimination in relation to AOD, and limited client insight into their underlying condition. Those health-related staff who had a special interest in homelessness and AOD use appeared to horizon scan more critically about these challenges of providing good palliative care to this population.

### Maintaining moral adequacy in the face of traumatic death

A hostel worker positioned her relationship with clients at the end of life by discussing how it had been within a context of limited family involvement:

I think you build a different relationship with someone when they don’t have their own family, because you want them to have that positive experience of their life. We sat with him [talking] about games consoles, TVs, pies, cream cakes, everything like that, stuff that family would do. So, when he passed away it was quite hard for the team. (Hostel worker FG 1)

The hostel worker re-categorizes a relationship with someone without family based on an ethical desire for positive life experiences for their client. She challenges the family membership categories of, for example, daughter/father^34,35^ and in the absence of these ties presents a list of activities to the audience in demonstrating how she has positioned herself in replicating positive familial activities. She finally presents the personal and emotional cost of such positionality in the face of the client’s death. This highlights the emotional labor that often occurs in developing meaningful relationships with clients who may have fractured familial ties, poor mental health and use AOD. Navigating often complex relationships and attempting to facilitate support can be morally challenging in the face of imminent dying, particularly if that dying is not managed well and if the resulting grief is not seen as legitimate by colleagues or family.

Most participants either implicitly or explicitly identified the ethical and emotional challenges of how to support people using AODs within wider social care. This ethical challenge was particularly acute when dying had not been recognized, the care needs and symptom management at the end of life had not been appropriately assessed, and the environmental and structural facilities did not meet the needs of the client. One hostel provider commented:

I get that we’ve got people there that are seriously ill and are on pathways themselves. But it puts us in a very, very difficult position in terms of what do we do with this individual?. . . So, we’re keeping somebody there, and we’re not able to fulfil somebody’s basic human needs. That’s very, very difficult, it’s very difficult for the staff and the management team, because we’re literally watching somebody possibly die in front of us, or in a lot of discomfort and pain because there isn’t the facilities out there to meet the needs of this individual. (Hostel manager FG 2)

The positioning of this dialogue between hostel and harm reduction workers explored the limited available alternatives to house people with complex needs and highlighted the structural and environmental context, which can contribute to a challenging end-of-life experience. The hostel worker within this dialogue accentuated the difficulty of supporting seriously ill clients as a collective team. She presents potential moral culpability by linking inadequate support to a violation of human rights. She again uses the adverb “very” three times to highlight the management challenges and focuses on the difficulty of watching someone die with appropriate symptom management. The conclusion of this narrative focused on inadequate care facilities to support end-of-life care within a hostel setting. However, this challenged the advocacy work required to access support, for example, another participant (a social worker) positions herself as an advocate to try to keep the client within a service that could support them.

Maintaining moral adequacy was positioned as requiring conversations with service providers across interdisciplinary lines and in advocating for clients. Participants identified a lack of acceptance by health and social care colleagues of some people’s desire to keep using AOD until death and this made supporting clients’ access to wider services more challenging. This often led to soul searching for health and social care practitioners, reflecting on whether outcomes could have been different and whether they had done enough.

Advocacy work for people using AODs can also be a difficult and challenging ethical practice. For example, some participants (particularly those working in social care), referred to mental capacity assessments within hospital settings and positioned hospital staff as assessing clients as having current mental capacity while the focus group participants often felt their clients did not have mental capacity to make decisions at that time. The actions by hospital staff often led to very quick discharge. This allowed for limited discharge preparation with interdisciplinary meetings potentially vital in ethically meeting the clients’ needs and facilitating safe discharge.

## Discussion

The significance of these data is in capturing the complexities of both patient/client needs and the subsequent moral distress this can precipitate with this minoritized and stigmatized population. Moral distress can be seen as occurring occasionally and potentially avoidable, but it can lead to moral injury, a consequence of constant moral distress and linked with poor mental health outcomes.^36^ The consequence of this moral distress may also be correlated to such negative concepts as burnout and compassion fatigue.^37,38^ For the participants of this study, maintaining moral adequacy required delivering effective care for their clients. In terms of a “good” death for homeless people, dying peacefully, not alone, with as much independence as possible within the dying phrase and surrounded by their social support network or family is important.^39^ As is being in a familiar environment and these factors were also a priority for people using AOD at the end of life.^40^ This remains a challenge given the complexity of care needs and the limited resources often available to hostel providers working with homeless people using AOD at the end of life.^41^ Ethically driven person-centered care was often compromised, not necessarily through the personal agency of practitioners but rather due to a wider professional, environmental and structural challenges. Such challenges left many practitioners isolated and ill-equipped to support end-of-life care for this population. This is particularly problematic when supporting unsheltered homeless people who have higher rates of mortality, stress and health co-morbidities compared to homeless people who are sheltered.^42^ Practitioners are often working with unsheltered homeless people with no clear prior medical history or diagnosis and frequently navigating situations of food and water insecurity.^43^ There are often complicated relationships with street-based friends with challenges regarding pain management in the presence of AOD.^12,44^ There remains a risk of drug diversion for prescribed opioids for end-of-life care, and difficulties with safe storage to prevent this.^45^ In the context of providing supervised consumption sites^46^ this again can present challenges for unsheltered homeless people using AOD. Engagement with this approach may be limited given other priorities related to food, water and environmental insecurities.

The empirical data drawn from the four focus groups in this study highlighted new data on the experiences practitioners had working with an AOD-using population with complex health, social care and housing needs at end of life. This complexity means that models of care require nuanced and responsive pathways that support and manage diverse multidisciplinary interventions for people using AOD at the end of life. Positioning theory^28^ provides a scaffold with which to explore different professional boundaries of practice and learning across health and wider social care. Good practice examples from existing evidence^11,47^ note there should be clear communication and joint working across interdisciplinary boundaries to best meet the needs of this vulnerable and often minoritized population. For example, Klop et al.^48^ evaluated joint training sessions, multi-disciplinary meetings and consultations between social service and palliative care professionals working with people who were homeless. They perceived an added value of the intervention in terms of collaboration, competences and quality and timing of palliative care. Positioning theory considers how everyday conversation involves teller and listener negotiating the construction(s) of actions relating to social practice, in this case, good practice across the three issues of homelessness, AOD use and end-of-life care.

These data suggest that practitioners have a more nuanced personal response in positioning and presenting moral adequacy in the face of “cracks of a ‘silo-ed’ care system” (Stajduhar et al.,^4^ p. 10) in which interdisciplinary communication is often poorly related to structurally disadvantaged populations.^1,41^ This built on the focus group data generated by Ebenau et al.,^49^ which also called for better interprofessional integration and collaboration. The challenge remains of how do practitioners navigate care in the face of such complexity given the organizational constraints and limited resources available while positioning themselves as moral and ethically competent? The detrimental psychological and existential challenges of moral distress for practitioners within palliative care have been extensively documented^50–52^ with some contributing factors including organizational constraints which contravene personal values. It remains important for staff to be aware and identify moral distress and while there is limited evidence of effective interventions^53^ generating educational materials and ongoing training could provide an avenue of support.^36^ This could include mechanisms to report moral distress and injury at an institutional level and how the organization and colleagues could facilitate supportive networks to effectively challenge stigma and discrimination. This could provide an environment where staff feel enough space and given “permission” to articulate the impact of moral distress and receive specialist support with organizational active monitoring programs to identify moral distress early.^54^

This study’s findings can be seen as both a need for organizational change and a resilience need for practitioners. The dilemma for workers is clearly coping with their personal and professional expectations of performance within a constrained and challenging environment, some of which may be seen and experienced as intractable and inflexible.

A scoping review by Perez et al.^55^ examining moral distress among practitioners working with homeless clients identified similar organizational and systems changes to mitigate the distress in working with this client group. They recommended managerial practices which included supervision, building informal supports, debriefing practices and instigating a range of strategies to create a healthy work environment. Geuenich et al.^56^ also identify supervision as a key intervention enabling people to identify, discuss and reflect on their experiences and in so doing, identify problem-solving strategies and personal coping. Importantly, they also indicate that regular supervision identifies moral distress and injury and therefore provides a form of early detection and intervention to support the practitioner.

Therefore, recommendations from our study focus on organizational changes to improve collaborative working and communication; this to deliver improved care and thus reduce moral distress. Also, however, there should be a focus on the workforce themselves by instigating occupational support practices such as clinical supervision (in person or teams), debriefing, work-life balance and strategies to demonstrate that staff are valued (such as bottom-up management consultations, rewards and informal support). There could also be induction for new staff working in homelessness that help identify and manage moral distress and highlight substance use and premature mortality among this client group.

Preferred place of care at the end of life was often discussed within the focus groups and this frequently troubled participants’ notions of a “good” death. Homelessness and AOD use challenged effective care at the end of life but also affected practitioner engagement with clients. Maintaining moral adequacy in delivering effective care became problematic when client self-care conflicted with practitioners’ perceived notions of what “self-care” entails and their professional priorities. There may be other multiple factors which also affect preferred place of care and support in dying, including stigma related to AOD use^57,58^ poor interdisciplinary coordination and understanding of structurally disadvantaged groups.^4,15^ This also includes the immediacy of daily living for this population preventing discussions related to advance care planning.^9^ These factors were positioned by participants as providing the context in which navigating ethical practice was so challenging. Galvani et al.^59^ refer to the “messy” picture often presented with more minoritized populations with complex care needs. Health and social care services are often not designed to meet these needs and the development of new models of care that bridge health and social care and address complexity is an important step to support ethical practice for practitioners.

### Limitations

There were several limitations to the study. First, data were collected during the COVID-19 pandemic, which may have been a contextual factor in how some of the participants responded, for example, non-return of demographic information. Only 9 of 24 participants returned demographic data thus limiting transparency of our sample profile. Multi-disciplinary working and support systems could have been altered or adapted, affecting the discourse of the focus groups and requiring some caution in interpreting these data post-pandemic. This study used purposive sampling, whereby participants volunteered to take part, potentially resulting in a selection bias, participants may not be representative of those who did not volunteer. Finally, all participants were sourced from the same region (North-West England). This can result in geographical bias, as the views may not be representative of practitioners working with homeless people nearing end of life in other areas.

## Conclusion

The originality and significance of data from this study are that working with people who are homeless and using substances at the end of life it is not just a challenge to professional risk and safety management but also, as Baruch^25^ describes, “the issue of their acceptance as moral persons, competent members and adequate performers. Hence, in formulating their accounts, they accomplish the status of moral adequacy” (p. 276). Within this study, practitioners positioned narratives and discourse within three inter-related areas; professional boundaries, maintaining moral adequacy and interpretations of a good death. These data suggest that maintaining moral adequacy was challenged through the complexity of needs people presented with, and the ability of formal services to address these needs. Ineffective multi-disciplinary working was also positioned as a factor in why people who are homeless using AOD at the end of life could receive poor care. This was partly driven by professional boundaries leading to siloed, isolated practice but also limited proactive planning required to facilitate good end-of-life care. Providing a “good” death and advance care planning were positioned by practitioners as particularly challenging since it was not always a priority for homeless people using AOD and, therefore, could lead to significant client and practitioner distress and poor symptom management at the end of life. Practitioners were exposed to client distress with limited ability to effectively manage the situations they were presented with. This ethical challenge remains difficult to address to maintain moral adequacy. It is, therefore, important to explore both the wider structural inequalities that can impede care and the interdisciplinary relationships that can better cooperate to enable more effective models of care. There is also further work required to expand the interventional evidence to mitigate the impact of moral distress in existing environmental contexts.
